# Uncovering Key Factors in Graphene Aerogel-Based Electrocatalysts for Sustainable Hydrogen Production: An Unsupervised Machine Learning Approach

**DOI:** 10.3390/gels10010057

**Published:** 2024-01-12

**Authors:** Emil Obeid, Khaled Younes

**Affiliations:** College of Engineering and Technology, American University of the Middle East, Egaila 54200, Kuwait

**Keywords:** graphene aerogel-based, electrocatalysts, hydrogen production, principal component analysis (PCA)

## Abstract

The application of principal component analysis (PCA) as an unsupervised learning method has been used in uncovering correlations among diverse features of aerogel-based electrocatalysts. This analytical approach facilitates a comprehensive exploration of catalytic activity, revealing intricate relationships with various physical and electrochemical properties. The first two principal components (PCs), collectively capturing nearly 70% of the total variance, attested the reliability and efficacy of PCA in unveiling meaningful patterns. This study challenges the conventional understanding that a material’s reactivity is solely dictated by the quantity of catalyst loaded. Instead, it unveils a complex perspective, highlighting that reactivity is intricately influenced by the material’s overall design and structure. The PCA bi-plot uncovers correlations between pH and Tafel slope, suggesting an interdependence between these variables and providing valuable insights into the complex interactions among physical and electrochemical properties. Tafel slope stands to be positively correlated with PC_1_ and PC_2_, showing an evident positive correlation with the pH. These findings showed that the pH can have a positive correlation with the Tafel slope, however, it does not necessarily reflect a direct positive correlation with the overpotential. The impact of pH on current density (*j*)and Tafel slope underscores the importance of adjusting pH to lower overpotential effectively, enhancing catalytic activity. Surface area (from 30 to 533 m^2^ g^−1^) emerges as a key physical property, inclusively inverse correlation with overpotential, indicating its direct role in lowering overpotential and increasing catalytic activity. The introduction of PC_3_, in conjunction with PC_1_, enriches the analysis by revealing consistent trends despite a slightly lower variance (60%). This reinforces the robustness of PCA in delineating distinct characteristics of graphene aerogels, affirming their potential implications in diverse electrocatalytic applications. In summary, PCA proves to be a valuable tool for unraveling complex relationships within aerogel-based electrocatalysts, extending insights beyond catalytic sites to emphasize the broader spectrum of material properties. This approach enhances comprehension of dataset intricacies and holds promise for guiding the development of more effective and versatile electrocatalytic materials.

## 1. Introduction

With the overreliance on non-renewable fossil fuels and the associated environmental concerns, the development of renewable energy sources becomes critical to mitigating environmental degradation and addressing global energy challenges [1]. In this instance, hydrogen (H_2_), serving as both a potent energy storage solution for intermittent renewables and a cleaner alternative to replace fossil fuels, stands as a pivotal element in our journey toward a more sustainable and environmentally conscious energy future [2,3].

In a larger sense, hydrogen generation typically follows two major pathways: one involves the use of electricity through electrolysis [4], commonly referred to as green hydrogen; the other involves reforming natural gas, which emits CO_2_. When CO_2_ is caught and permanently stored, the resulting hydrogen is sometimes referred to as blue hydrogen [4]. As a result, electrochemical water splitting (ECWS) is recognized as an efficient and environmentally friendly method for hydrogen production, contributing significantly to the achievement of the United Nations Sustainable Development Goals (SDGs) 7 (Affordable and Clean Energy) and 13 (Climate Action) [2,4]. The theoretical minimum thermodynamic voltage needed for electrochemical water splitting (ECWS) at 80 °C is 1.229 V, commonly known as overpotential [5,6].

Several studies have looked into ways to reduce overpotential, such as functioning in acidic or basic environments [3,7,8,9]. Notably, Caravaca et al. [5] developed a reactor based on polymer electrolyte membranes that was used for continuous-flow electrolysis of lignin-based alkaline solutions. In comparison to conventional water electrolysis, this novel design allows hydrogen production at the cathode with substantially lower overpotential, starting at around 0.45 V.

Another strategy for reducing overpotential is to utilize electrocatalytic and photocatalytic materials as cathode and anode to improve water-splitting efficiency. Current electrocatalysts are often made of noble metals due to their low overpotential and high current density [5,6]. Nevertheless, the widespread use of these catalysts is hampered by their high pricing and limited availability [10]. As a result, creating cost-effective, plentiful, and highly efficient electrocatalytic materials to accelerate the water-splitting process becomes critical [11]. Chatenet et al. [12] conducted a thorough study of the fundamental elements of electrocatalytic water splitting, giving insights from both academic and institutional research as well as large-scale industrial processes. Significant progress has been made in the field of nanostructured electrocatalysts during the last decade, thanks to both theoretical and experimental studies. These efforts have resulted in significant advancements in the production of electrocatalysts such as carbons, polymers, ceramics, and metals [8,9,13]. This advancement has been made possible by carefully regulating the shape, electrical properties, and surface features of the nanomaterials [14,15,16]. Electrocatalysts based on the use of three-dimensional (3D) porous nanostructured materials have a lot of potential in this field. Their improved electrocatalytic efficiency is due to subtleties embedded within the catalyst’s design, stressing that the efficacy of these materials is dependent on the overall configuration and structural properties of the catalyst [17,18,19]. According to research findings, three-dimensional (3D) porous architecture, which is created through the assembly of molecular precursors or low-dimensional nanostructures, not only includes intrinsic properties derived from its confined dimensions but also demonstrates emergent properties. These include a large interior surface area and the promotion of efficient molecule transport, emphasizing the numerous benefits inherent in such constructed structures [19,20]. The three-dimensional catalyst is gaining attention, indicating its importance not only in the field of hydrogen production but also in the equally essential field of soot oxidation [21,22]. The unique three-dimensional design provides a significant improvement, playing a critical role in improving the efficacy and performance of the catalyst in hydrogen production, soot oxidation, and many other applications [23,24,25]. This demonstrates the three-dimensional catalyst architecture’s adaptability and significant promise in enhancing numerous aspects of catalytic processes.

Aerogels have received a lot of interest for their unique physicochemical properties in the roles of catalysts and catalyst supports for both cathodes and anodes. These include large surface areas, an open meso- and macroporous structure, configurable surface chemistry, and an extraordinarily lightweight density [20]. Aerogel-based catalysts have a number of advantages. Aerogels can be treated with a variety of precursors or building blocks, each with unique features, to tune certain catalytic attributes and achieve improved performance. Furthermore, these aerogels can act as effective supports, allowing for the loading of diverse precursors, increasing their catalytic performance [26]. Second, the self-supporting 3D porous structure in aerogel monoliths is particularly appealing because the catalyst benefits from the unique intrinsic properties of active materials, and the interconnected meso- and macropores facilitate multidimensional electron and ion transport pathways within the 3D network, increasing catalytic efficiency [20,27]. The monolithic 3D porous property of aerogel catalysts typically mitigates the inhomogeneous agglomeration and re-stacking difficulties encountered with low-dimensional-based catalysts, resulting in the loss of surface area and catalytic activity during ECWS. Finally, because redox reactions frequently occur at the surface and interfaces of materials, the vast surface area of aerogels can provide a large number of active sites for redox reactions [19]. Due to the versatility of aerogel synthesis and the ability to use alternative building blocks, the active catalytic sites can be easily modified by altering the surface chemistry. Foams, which are similar to aerogels in most characteristics, are another highly researched FPM. Foams, like aerogels, have macroscopically very porous monolithic structures that make handling and applications easier. Furthermore, foams can be made from a variety of materials, including silica, carbons, polymers/biopolymers, ceramics, and two-dimensional (2D) materials, as well as inorganic nanocrystals [28,29,30,31].

Numerous studies have been conducted to increase the catalytic effectiveness of aerogel-based electrocatalysts in the water-splitting reaction, with the goal of lowering the related overpotential [20,26,32]. Several parameters, including pH settings, electrocatalyst surface area, catalytic loading on various types of aerogels, Tafel slope, and electrolyte type, were carefully considered to achieve this goal [3,5,20,26,32,33]. These characteristics were thoroughly investigated, but their intercorrelations remained unknown, preventing a comprehensive analysis that may provide useful insights into the problem of large overpotentials. As a result, this study introduces the use of principal component analysis (PCA) as an unsupervised learning method to investigate the interrelationships between different features of aerogel-based electrocatalysts, basing on the review of Al-Hamamre et al. [3]. Furthermore, based on the PCA results, critical parameters impacting the catalytic activity of several aerogel-based electrocatalysts for the water-splitting reaction were found by studying the correlation between these electrocatalysts’ characteristics and their related overpotential.

This work reports that the cumulative contribution of the first two principal components (PC_1_ and PC_2_) accounted for 69.15% of the total variance. Examining the variables within PC_1_, pH, surface area, and Tafel slope demonstrated significant contributions. Notably, overpotential and catalyst loading exerted minor influences on PC_1_. While catalyst loading exhibited a negligible impact on PC_1_, its highest contribution emerged in PC_2_, accounting for nearly 52%. A notable exception was the close proximity of pH and Tafel slope, indicating a discernible positive correlation between these two variables within the scope of the investigated materials. These findings indicate a positive correlation between pH and Tafel slope, suggesting that changes in pH may influence Tafel slope. However, the lack of a direct positive correlation between pH and overpotential highlights the complexity of the relationship. This complexity is attributed to the linear relationship between current density and pH, where a decrease in pH dramatically reduces current density, increasing the activation energy of electron transport [33]. Another PCA finding suggests that increasing the surface area is a significant factor directly associated with lowering the overpotential. The intricate relationship between surface area and overpotential, as unveiled by our analysis, emphasizes the importance of considering this specific physical property in the design and optimization of aerogel-based electrocatalysts to enhance their overall performance.

## 2. Results and Discussion

The application of principal component analysis (PCA) as an unsupervised learning method was employed to explore the correlation among various features of aerogel-based electrocatalysts. The data analyzed in this study were compiled from the comprehensive review conducted by Al-Hamamre et al. [3]. Our analysis is based on the overpotential as a reference to discuss the catalytic activity and its relation with different physical and electrochemical properties of various aerogel-based electrocatalysts as reported by Al-Hamamre et al. [3].

**Figure 1:**
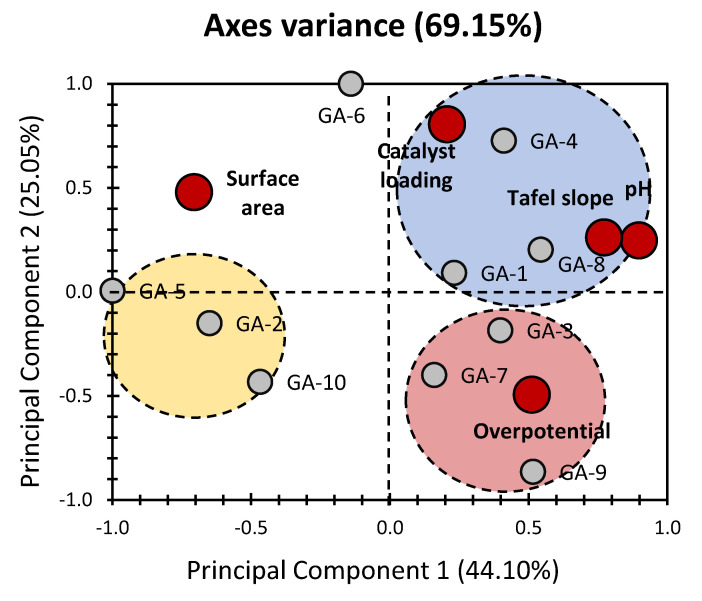
PC_1_ vs. PC_2_ representation of datasets for the properties of aerogel-based electrocatalysts (data were obtained from the previous findings of Al-Hamamre et al. [3]). Grey bullets indicate the different electrocatalysts under investigation (GA: graphene aerogels). Red bullets indicate physical properties of electrocatalysts (physical and electrochemical properties).

**Figure 2:**
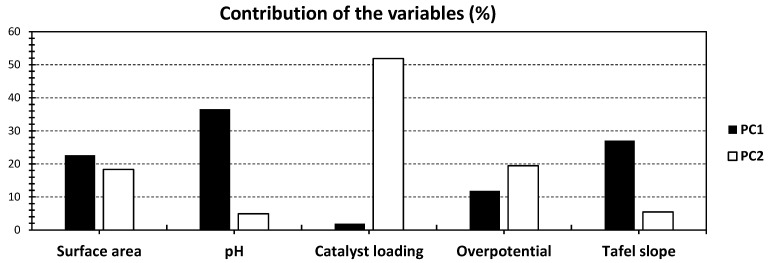
% contribution of the different variables of Figure 1, relative to PC_1_ (black) and PC_2_ (white).

Figure 1 illustrates the results of principal component analysis (PCA) examining the physical and electrochemical properties of various graphene aerogel-based electrocatalysts as reported by Al-Hamamre et al. [3]. The cumulative contribution of the first two PCs accounts for 69.15% of the total variance, with PC_1_ and PC_2_ representing 44.10% and 25.05%, respectively (Figure 1). Analyzing the variables within PC_1_, pH stands out with a significant contribution of 36.53%, followed by moderate contributions from surface area (22.62%) and Tafel slope (27.06%) (Figure 2). Notably, overpotential and catalyst loading exert minor to negligible influences on PC_1_. The pronounced impact of pH on the electrocatalysts suggests the substantial influence of electrolyte concentration and type (acid or base) on catalytic activity in the investigated materials [1]. It is noteworthy that while the catalyst loading exhibits a negligible influence on PC_1_, its highest contribution emerges in PC_2_, accounting for nearly 52% (as depicted in Figure 2). The remaining investigated variables show minor to negligible influences on PC_2_. These diverse findings imply that the catalyst loading does not exhibit a major impact on the overall physical, chemical, and electrochemical features of the material. This suggests that the development of a potential catalytic material extends beyond the consideration of catalyst loading to encompass various features of the material under investigation, including its textural, chemical, and electrochemical properties in order provide a large number of active sites for redox reactions. Previous studies have highlighted the multifaceted nature of these matrices in influencing catalytic efficiency [3], reinforcing the need for a comprehensive understanding of the intricate interplay of factors in catalytic material design and processing. In accordance with this finding, Obeid et al. [34] demonstrate the critical involvement of the bulk oxygen species of yttria-stabilized zirconia (YSZ) in the soot oxidation process. Assume that the soot oxidation on YSZ is initiated by an electrochemical mechanism comparable to that of a fuel cell at the nanometric scale. This electrochemical process’s efficiency appears to be determined by both the YSZ/soot interaction where the design of YSZ plays a crucial role, i.e., open porosity with 3DOM structure [21,22].

The PCA bi-plot perceptibly distributes the five investigated variables, creating a visual representation of their relationships. Interestingly, a notable exception lies in the close proximity of pH and Tafel slope on the plot, suggesting a discernible positive correlation between these two variables within the scope of the investigated materials. This proximity highlights the potential interdependence of pH and Tafel slope, indicating that changes in one variable may be associated with corresponding changes in the other. Such correlations provide valuable insights into the intricate interactions and dependencies among the physical and electrochemical properties of the aerogel-based electrocatalysts [3]. These findings are in total accordance with the investigations of Bao et al. [7], showing that higher pH increases the Tafel slope. Tafel slope is a measurement of how well an electrode generates current in response to changes in applied potential [35,36]. Al-Hamamre et al. [3] reported that a lower Tafel slope implies that less overpotential is required to obtain a high current density, implying faster electrocatalytic reaction kinetics. The current density is a broad parameter that defines the intrinsic charge transfer in equilibrium [35,36]. A high j value indicates that electron transport is simple with a low activation energy [35,36]. Zalitis et al. [33] demonstrate the pH negative dependency of the logarithm of exchange current density in the electrolytes from pH 0.6 to 1. In this study, the exchange current density showed an approximately linear relationship with pH [33]. The latter explains that a higher pH results in a lower logarithm of exchange current density (log(*j*)) (when electron transport is difficult and activation energy is high) and therefore higher Tafel slope [33]. However, Figure 1 shows that the overpotential stands to be positively correlated with PC_1_ and negatively correlated with PC_2_. On the other hand, the Tafel slope stands to be positively correlated with PC_1_ and PC_2_ which shows an evident positive correlation with the pH. These findings showed that the pH can have a positive correlation with the Tafel slope, however, it does not necessarily reflect a direct positive correlation with the overpotential. The latter is due to the linear relationship between the current density and the pH, where the pH can dramatically decrease the current density and therefore increase the activation energy of electron transport as reported by Zalitis et al. [33]. This finding suggests that lowering the overpotential (increasing the catalytic activity) is contingent upon the influence of pH on current density. It is crucial to adjust the pH appropriately to avoid diminishing current density, thereby mitigating the undesirable consequence of increasing the activation energy required for electron transport.

Within the assortment of diverse materials examined in the PCA, a discernible pattern emerges, leading to the identification of three distinct clusters denoted by Blue, Red, and Yellow (Figure 1). This clustering, coupled with the substantial variance observed, underscores the efficacy of PCA in unveiling intriguing patterns and relationships among the investigated materials. The Blue cluster, situated on the positive side of both PCs, incorporates materials GA-1, GA-4, and GA-8. The cluster demonstrates a positive correlation with catalyst loading, Tafel slope, and pH. This correlation shows that enhancing these attributes could improve material performance by lowering the overpotential, when the pH influence on current density can be controlled suitably, within this particular cluster. In contrast, the Red cluster, comprising GA-3, GA-7, and GA-9, is located on the positive and negative sides of PC_1_ and PC_2_, respectively. This cluster exhibits a pronounced positive correlation exclusively with overpotential. However, the surface area is negatively correlated with respect to the latter. This implies that these materials may necessitate a higher electrical potential to operate more efficiently. The Yellow cluster, encompassing GA-2, GA-5, and GA-10, positions itself on the negative side of PC_1_, displaying a negligible to moderate negative correlation with PC_2_. Unlike the Blue and Red clusters, no substantial positive correlation with any variable is observed for the Yellow cluster. However, moderate influences from surface area along the negative side of PC_1_ and overpotential along the negative side of PC_2_ are noteworthy. This suggests that the materials in the Yellow cluster manifest unique behavior compared to other investigated materials, emphasizing the need for a more comprehensive set of properties to uncover the significant influencers of these materials.

In essence, the distinct clusters not only elucidate varied material behaviors but also prompt a deeper exploration to unravel the underlying factors contributing to their unique characteristics. GA-2, GA-5, and GA-10 possess a relatively large surface area (>294 m^2^ g^−1^) among various aerogel-based electrocatalysts as reported by Al-Hamamre et al. [3]. Therefore, based on the negative correlation between the surface and overpotential, GA-2, GA-5, and GA-10 should have the lowest overpotential, which is not the case. This result may be explained based on their lowest catalyst loading (<280 μg.cm^−2^). In addition, GA-2, GA-5, and GA-10 have the lowest pH and, therefore, based on the PCA results should have the lowest Tafel slope, however, this does not necessarily lead to the lowest overpotential, as previously discussed. These findings are very consistent with the datasets obtained from the findings of Al-Hamamre et al. [3]. GA-6 has been single-handedly plotted as having the highest positive influence for PC_2_, with a negligible influence on PC_1_. This would indicate the intricate influence of the catalytic loading for this material (Figure 1).

On the other hand, Figure 1 shows that the overpotential stands to be positively correlated with PC_1_ but negatively correlated with PC_2_. However, the only physical property that showed an inclusive opposite correlation (positively correlated with PC_2_ but negatively correlated with PC_1_) is the surface area (Figure 1). Therefore, the increase in the surface area is one of the major factors that may directly lower the overpotential.

**Figure 3:**
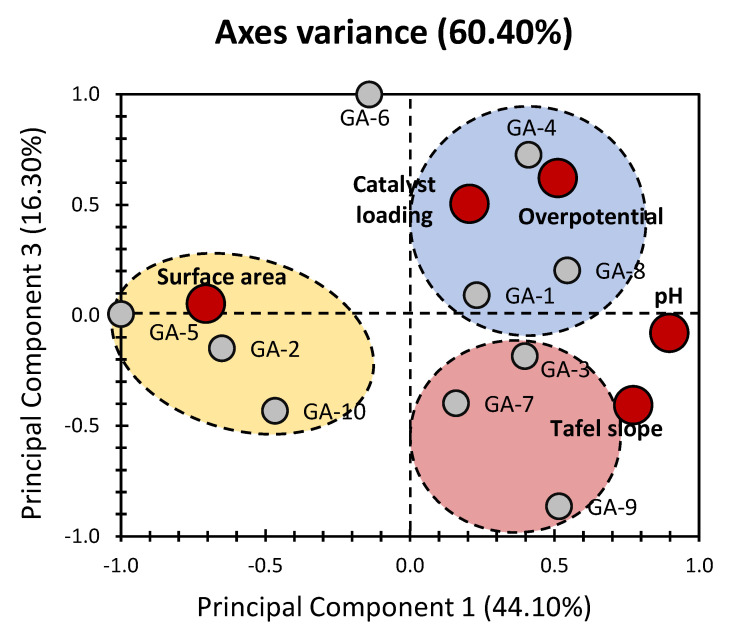
PC_1_ vs. PC_3_ representation of datasets for the properties of aerogel-based electrocatalysts (data were obtained from the previous findings of Al-Hamamre et al. [3]). Grey bullets indicate the different electrocatalysts under investigation (GA: graphene aerogels). Red bullets indicate physical properties of electrocatalysts (physical and electrochemical properties).

**Figure 4:**
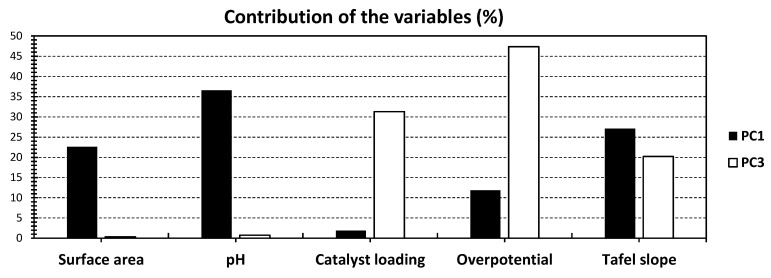
% contribution of the different variables of Figure 3, relative to PC_1_ (black) and PC_3_ (white).

To further unveil correlations within the dataset, the introduction of PC_3_ is incorporated in Figure 3 and Figure 4. The PCA bi-plot of PC_1_ and PC_3_ reveals a combined 60.40% of the total variance, with PC_3_ accounting for 16.30% (Figure 3). Despite achieving a slightly lower variance through these axes’ combination, the obtained variance remains acceptable, providing valuable insights into the dataset. Notably, the variables exhibit a clearer distinction across the investigated PCs. Each variable shows a singular contribution for either of the investigated PCs, except for Tafel slope, which scores nearly 27% and 20% for PC_1_ and PC_3_, respectively.

Examining the influence of each variable, overpotential emerges as the predominant factor for PC_3_, contributing the highest percentage at 47.39% (Figure 4). Catalyst loading also exhibits a moderate contribution of 31.29% (Figure 4). While the introduction of PC_3_ results in lower variance, it enhances the visualization of dataset patterns, offering a more refined understanding of the relationships between variables. In terms of correlations between variables, the previously observed high positive correlation between Tafel slope and pH persists. Additionally, an intriguing correlation between catalyst loading and overpotential is noted.

When considering the distribution of different materials (individuals), a consistent pattern is observed, validating the findings from the initial presentation (Figure 1). This suggests a high similarity between materials within the same cluster, reaffirming the trends identified in the previous approach. The persistent clustering pattern emphasizes the reliability of the identified trends and correlations, reinforcing the robustness of the analytical approach in capturing meaningful relationships within the intricate electrochemical dataset.

## 3. Conclusions

The application of principal component analysis (PCA) as an unsupervised learning method has proven to be instrumental in unraveling the correlation among various features of aerogel-based electrocatalysts. This analytical approach facilitates a comprehensive discussion of catalytic activity and its intricate relationship with different physical and electrochemical properties, as extensively reported by Al-Hamamre et al. [3]. The first two principal components (PCs) collectively capture nearly 70% of the total variance. This substantial coverage attests to the reliability of the approach and its efficacy in revealing meaningful patterns from the dataset. Intriguingly, a material’s reactivity is not primarily dictated by the amount of catalyst loaded; rather, it is delicately sculpted by the material’s overall design and structure. These findings contradict the dominant belief that the catalysts’ loading would exhibit a major influence on the catalytic activity and behavior. Other parameters, such as textural features, can considerably contribute to the formation of active sites and, as a result, increase catalytic activity.

The PCA bi-plot shows correlations between pH and Tafel slope underscore a potential interdependence between these two variables, indicating that changes in pH may be associated with corresponding alterations in the Tafel slope. These insights offer valuable understanding of the intricate interactions and dependencies among the physical and electrochemical properties of aerogel-based electrocatalysts. The findings further highlight the intricate relationship between overpotential and principal components (PC_1_ and PC_2_) as opposed to a direct positive correlation with pH. This underscores the complex impact of pH on current density and, consequently, the activation energy of electron transport. Hence, there is a crucial role of appropriately adjusting pH to prevent the undesirable consequences of reduced current density and increased activation energy, ensuring effective control over overpotential and enhancing catalytic activity. In addition, the surface area emerges as the singular physical property demonstrating an inclusive opposite correlation—positively correlated with PC_2_ while negatively correlated with PC_1_. This finding underscores the significance of surface area as a major contributing factor that may directly lower the overpotential. The inverse relationship between surface area and overpotential suggests that an increase in surface area is associated with a potential reduction in overpotential and therefore increases the catalytic activity.

To delve deeper into the correlations within the dataset, the introduction of PC_3_, in conjunction with PC_1_, enriches the analysis. Despite a marginally lower variance (60%), this combination reveals trends similar to the first two PCs, providing a clearer distinction among the variables. The consistency in trends between this approach and the initial one not only validates the robustness of PCA but also underscores its applicability in delineating the distinct characteristics of graphene aerogels and affirming their potential implications in diverse electrocatalytic applications.

In brief, the employment of PCA in this study has proven to be a valuable tool for unraveling the complex relationships among various features of aerogel-based electrocatalysts. The different insights gained from this analysis extend our understanding beyond the mere consideration of catalytic sites, emphasizing the importance of evaluating the broader spectrum of material properties. This approach not only enhances our comprehension of the intricacies within the dataset but also holds promise for guiding the development of more effective and versatile electrocatalytic materials.

## 4. Materials and Methods

### 4.1. Data Collection and Normalization

Data have been gathered from the study conducted by Al-Hamamre et al. [3]. Table 1 provides an overview of the various materials under investigation, including their pH, surface area, overpotential, Tafel slope, and catalyst loading. Each variable included in the study is unique regarding the material, following several applications [3]. To address potential biases stemming from differences in magnitudes, a normalization technique similar to the one employed by Murshid et al. [37] was utilized.

(1) $$Y_{st}=\frac{\left(Value-Mean\right)}{StandardDeviation}$$

where “*Y_st_*” presents the standardized dataset values.

**Table 1:** Physical and electrochemical properties of different graphene aerogel-based electrocatalysts.

Catalyst		Surface Area (m^2^ g^−1^)	pH	Catalyst Loading (µg cm^−2^)	Overpotential (mV)	Tafel Slope (mV dec^−1^)	References
Ni_3_FeN/graphene aerogel	GA-1	171	14	500	94	90	[38]
CoP/graphene aerogel	GA-2	532.2	0.1	280	121	61	[39]
Ru/N-graphene aerogel	GA-3	244.8	13	100	145	109	[40]
(Ni,Co)Se_2_/graphene aerogel	GA-4	123	14	2650	128	79	[41]
Co-N-graphene aerogel	GA-5	466.6	0.1	275	50	33	[42]
MoS_2_/graphene aerogel	GA-6	700	14	2000	120		[43]
CoP-C/graphene aerogel	GA-7	31.4	14	280	120	57	[44]
WSe_2_/NiFe- LDH/N,S-graphene aerogel	GA-8	110	14	1000	122	112	[45]
CoP-C/graphene aerogel	GA-9	31.4	14	280	225	66	[44]
MoS_2_/graphene aerogel	GA-10	294	0.1		162	41	[46]

### 4.2. Principal Component Analysis (PCA)

After applying normalization, the PCA outcomes were derived through the use of XLSTAT 2014 software (16.5.03), following a methodology in alignment with the procedure described by Younes et al. [47]. To handle any missing data in this investigation, a built-in feature was employed, substituting the unavailable values with the “mode” based on the relevant variables.

The study’s objective is to employ PCA on data from a prior study by Al-Hamamre et al. [3] (Table 1). PCA serves as a powerful tool aimed at unveiling intricate patterns existing between the bulk properties of the examined materials and the specific characteristics of aerogels [47,48]. This endeavor significantly contributes to the interpretation and comprehension of the factors dictating the suitability of aerogels in several applications [47]. By delving into the hidden patterns, the PCA output provides valuable insights into the broader implications for aerogel applications, encompassing considerations such as chemical, physical, and even textural properties of the materials at hand [37,47].

The complexity of the analysis lies in the incorporation of five distinct factors influencing the behavior of 10 investigated aerogels, as outlined in Table 1. Operating as a data-driven, unsupervised machine learning technique, PCA strategically reduces the dataset, leading to improved visualization and facilitating the revelation of concealed patterns through correlations, whether negative or positive. The representability of principal components (PCs) in relation to the overall population further enriches the analysis, offering an understanding of the interplay between various factors influencing the performance of aerogels’ applications. The *j*^th^ PC matrix (*Fi*) is expressed using a unit-weighting vector (*U_j_*) and the original data matrix *M* with *m* × *n* dimensions (*m*: number of variables, *n*: number of datasets) as outlined [49,50,51]. The mathematical approach of PCA is as follows:

(2) $$Fi=U_{j}^{T}M=\sum_{i=0}U_{ji}M_{i}$$

where *U* is the loading coefficient and *M* is the data vector of size *n*. The variance matrix *M*(*Var*(*M*)), obtained by projecting *M* to *U*, should be maximized, following:

(3) $$Var\left(M\right)=\frac{1}{n}\left(UM\right){\left(UM\right)}^{T}=\frac{1}{n}{UMM}^{T}U$$

(4) $$MaxVar\left(M\right)=Max\left(\left(\frac{1}{n}\right)\;{UMM}^{T}U\right)$$

Since $\frac{1}{n}{MM}^{T}$ is the same as the covariance matrix of *M*(*cov*(*M*)), *Var*(*M*) can be expressed, following:

(5) $$Var\;\left(M\right)=U^{T}cov\;\left(M\right)\;U$$

The Lagrangian function can be defined by performing the Lagrange multiplier method, following:

(6) $$L=U^{T}$$

(7) $$L=U^{T}cov\left(M\right)U-\delta (U^{T}U-I)$$

For (7), “*U^T^U*−*I*” is considered to be equal to zero, since the weighting vector is a unit vector. Hence, the maximum value of *var*(*M*) can be calculated by equating the derivative of the Lagrangian function (*L*), with respect to *U*, following:

(8) $$\frac{dL}{dU}=0$$

(9) $$cov\left(M\right)U-\delta U=\left(cov\left(M\right)-\delta I\right)U=0$$

where *δ*: eigenvalue of *cov*(*M*); U: eigenvector of *cov*(*M*).

## Data Availability

The data presented in this study are openly available in article.
